# Transcriptional regulation of human amelotin gene by interleukin‐1β

**DOI:** 10.1002/2211-5463.12434

**Published:** 2018-05-14

**Authors:** Mizuho Yamazaki, Masaru Mezawa, Keisuke Noda, Yasunobu Iwai, Sari Matsui, Hideki Takai, Yohei Nakayama, Yorimasa Ogata

**Affiliations:** ^1^ Departments of Periodontology Nihon University School of Dentistry at Matsudo Japan; ^2^ Research Institute of Oral Science Nihon University School of Dentistry at Matsudo Japan

**Keywords:** amelotin, gene promoter, inflammation, interleukin‐1β, junctional epithelium, periodontitis

## Abstract

One of the major causes of tooth loss is chronic inflammation of the periodontium, the tissues surrounding the tooth. Amelotin (AMTN) is a tooth enamel protein which is expressed in maturation‐stage ameloblasts and also in the internal basal lamina of junctional epithelium, a unique epithelial structure attached to the tooth surface which protects against the constant microbiological challenge to the periodontium. Localization of AMTN suggests that its function could be involved in the dentogingival attachment. The purpose of this study was to investigate the effect of interleukin‐1β (IL‐1β) on AMTN gene transcription in human gingival epithelial Ca9‐22 cells. IL‐1β increased AMTN mRNA and protein levels at 3 h, and the levels reached maximum at 6 and 12 h. IL‐1β induced luciferase activities of human AMTN gene promoter constructs (−211, −353, −501, −769, and −950AMTN), but these activities were partially inhibited in −353AMTN constructs that included 3‐bp mutations in CCAAT/enhancer binding protein 1 (C/EBP1), C/EBP2, and Ying Yang 1 (YY1) elements. Transcriptional activities induced by IL‐1β were abrogated by protein kinase A (PKA), tyrosine kinase, mitogen‐activated protein kinase kinase (MEK1/2), and phosphatidylinositol 3‐kinase (PI3K) inhibitors. Gel shift and ChIP assays showed that IL‐1β increased C/EBPβ binding to C/EBP1 and C/EBP2, and YY1 binding to YY1 elements after 3 h, and that these DNA–protein interactions were inhibited by PKA, tyrosine kinase, MEK1/2, and PI3K inhibitors. These results demonstrated that IL‐1β increases AMTN gene transcription in human gingival epithelial cells mediated through C/EBP1, C/EBP2, and YY1 elements in the human AMTN gene promoter.

**Abbreviations**
AMTN: amelotin
AP1: activator protein 1
C/EBP: CCAAT/enhancer binding protein
HA: herbimycin A
IL‐1β: interleukin‐1β
JE: junctional epithelium
PI3: phosphatidylinositol 3
PKA: protein kinase A
PKC: protein kinase C
YY1: Ying Yang 1

Periodontitis is a chronic inflammatory disease and one of the major causes of tooth loss 1. The bacterial plaque attached to the tooth causes gingival inflammation that proceeds to damage the periodontium 2. Periodontopathic bacteria are one of the risk factors for periodontitis, and they induce immune responses and secretion of proinflammatory cytokines 3.

Interleukin‐1β (IL‐1β) is an inflammatory cytokine with a wide range of biological activities and produced by various types of cells. It is involved in cell proliferation, differentiation, apoptosis, and in the pathophysiology of periodontitis 4. The inflammatory responses mediated by IL‐1β play an important role in periodontal tissue destruction 5. IL‐1β‐dependent mechanisms may contribute to the inflammation and destruction of bone and attachment loss, which are characteristic features of periodontal disease 6.

Junctional epithelium (JE) is a unique epithelial structure located at the bottom of gingival sulcus and seals off the supporting tissues of the tooth from the constant microbiological challenge. JE is attached to tooth surface by hemi‐desmosome and represents the first line of defense against periodontal disease. This incompletely differentiated nonkeratinizing epithelium is formed by the fusion of reduced enamel organ with oral epithelium 7, 8, 9, 10.

Amelotin (AMTN) was initially identified in maturation‐stage ameloblasts as an ameloblast‐specific gene 11. Expression profiling revealed that AMTN protein is produced by maturation‐stage ameloblast and also expressed in the internal basal lamina of JE 12, 13. In terms of amelogenesis, AMTN‐overexpression mice showed thin and disorganized tooth enamel compared to wild‐type mice 14, and heavy erosion and attrition of mandibular incisors were observed in AMTN‐knockout mice 15. AMTN could induce hydroxyapatite mineralization 16, 17, and it is essential for proper enamel maturation 18. Function of the AMTN in JE has not yet been clarified; however, it is presumed that the localization of AMTN could be involved in attachment between JE and tooth enamel 19, 20. Odontogenic ameloblast‐associated protein (ODAM) and follicular dendritic cell‐secreted protein (FDC‐SP) are other components of the internal basal lamina of JE 21, 22. After gingivectomy, ODAM was detected first at the leading wound edge and then throughout the cells of the long JE. AMTN appeared later, and it was observed only at the cell–tooth interface 23. In this study, we used human gingival epithelial Ca9‐22 cells. They have similar characteristics to those of JE‐derived cells, because they express AMTN and FDC‐SP, which are components of the internal basal lamina of JE 22, 24.

We have previously reported that AMTN gene expression was increased in inflamed gingiva in patients with chronic periodontitis 24, 25, 26. Therefore, the regulation of AMTN gene transcription by IL‐1β in gingival epithelial cells is a crucial topic for onset and progression of periodontitis.

In this study, we focused on the expression of AMTN in gingiva and investigated the effects of IL‐1β on AMTN gene expression in human gingival epithelial cells.

## Results

### Effects of IL‐1β on AMTN mRNA and protein expression

**Figure 1:**
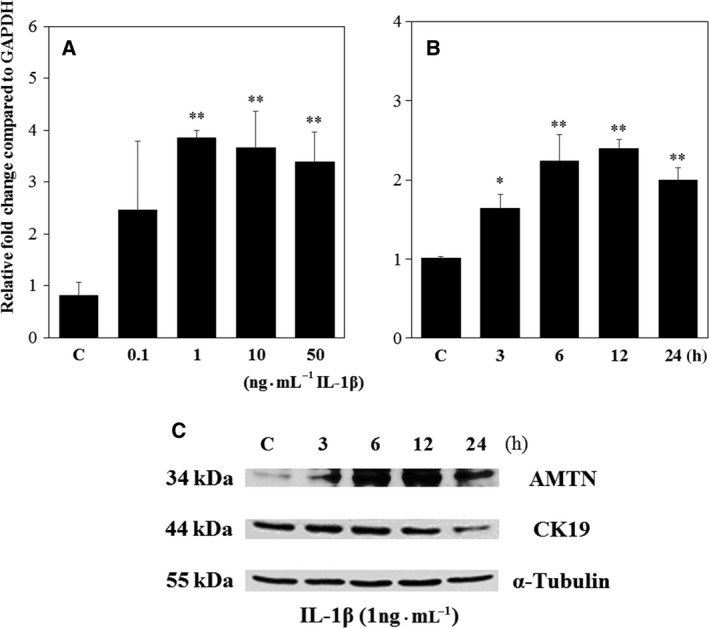
Effects of IL‐1β on AMTN mRNA and protein levels in Ca9‐22 cells. (A) Dose–response effects of IL‐1β on AMTN mRNA levels in Ca9‐22 cells treated for 12 h. (B) Ca9‐22 cells were treated with or without IL‐1β (1 ng·mL^−1^) for 3, 6, 12, and 24 h. AMTN and GAPDH mRNA levels were analyzed by real‐time PCR. The experiments were performed in triplicate for each data point. Quantitative analyses of the data sets are shown with standard errors. Significantly different from control, *P < 0.05 and **P < 0.01. (C) AMTN protein levels in Ca9‐22 cells were analyzed by western blotting using anti‐AMTN, anti‐CK19, and anti‐α‐tubulin antibodies.

To study the regulation of AMTN gene transcription by IL‐1β, we performed real‐time PCR using total RNA obtained from Ca9‐22 cells. The dose–response relation of AMTN mRNA levels after stimulation by IL‐1β was established by treating Ca9‐22 cells with different concentrations of IL‐1β for 12 h. IL‐1β increased AMTN mRNA expressions at 1, 10, and 50 ng·mL^−1^ (Fig. 1A). Thus, 1 ng·mL^−1^ IL‐1β was used to study the time‐course effect on AMTN mRNA levels. IL‐1β (1 ng·mL^−1^) induced AMTN mRNA levels at 3 h, and the levels reached maximum at 6 and 12 h (Fig. 1B). AMTN protein levels were increased by IL‐1β (1 ng·mL^−1^) at 3 h, reached maximum at 6 and 12 h, and decreased at 24 h. Cytokeratin 19 (CK19) was used as a marker of epithelial cells. IL‐1β (1 ng·mL^−1^) decreased CK19 protein levels at 24 h. α‐Tubulin was used as a loading control (Fig. 1C).

### Immunofluorescence

**Figure 2:**
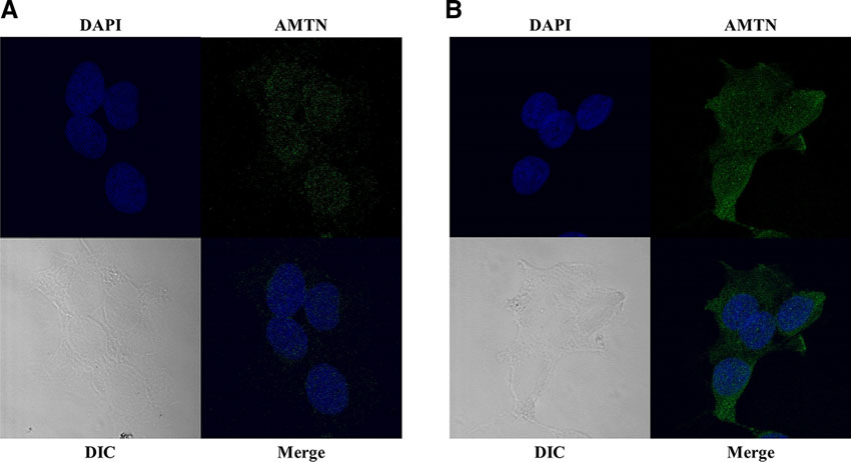
Immunofluorescence of the expression of AMTN in Ca9‐22 cells. Ca9‐22 cells were cultured in α‐MEM with 10% FBS for 12 h and changed to α‐MEM without serum for 6 h, and then, cells were treated without (A; control) or with 1 ng·mL^−1^ IL‐1β for 6 h (B). Nuclei and AMTN were stained with DAPI (blue) and anti‐AMTN antibody through immunofluorescence via a secondary antibody bound to Alexa Fluor 488 (green). Nuclei and AMTN expression in Ca9‐22 cells appeared in the merged image (×100). DIC was used for gaining proper images of unstained cells.

Immunofluorescence of the expression of AMTN in Ca9‐22 cells was increased after stimulation by 1 ng·mL^−1^ IL‐1β for 6 h (B) compared with Ca9‐22 cells without treatment by IL‐1β (A; control). Nuclei and AMTN were stained with DAPI (blue) and anti‐AMTN antibody via a secondary antibody bound to Alexa Fluor 488 (green). Nuclei and AMTN expressions in the cells appeared in the merged image (×100). Differential interference contrast (DIC) was used for gaining proper images of unstained cells (Fig. 2).

### Luciferase assays using human AMTN gene promoter constructs

**Figure 3:**
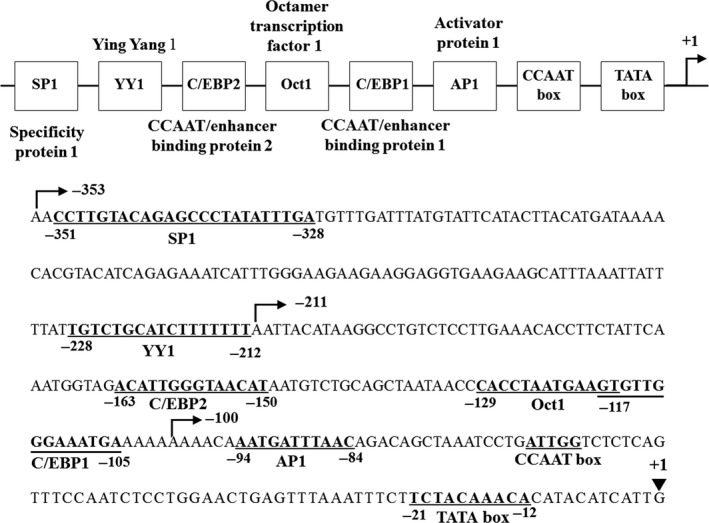
Regulatory elements in the proximal promoter of the human AMTN gene. Upper panel: The schematic diagram of human AMTN gene proximal promoter. Lower panel: The nucleotide sequences of the human AMTN gene promoter encompassing an inverted TATA box, inverted CCAAT box, AP1, C/EBP1, Oct1, C/EBP2, YY1, and SP1 are shown from ‐353 to transcription start site.

**Figure 4:**
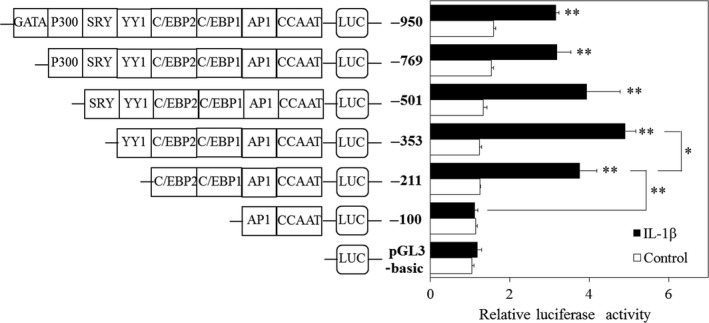
IL‐1β upregulates human AMTN gene promoter activities. The transcriptional activities of −211AMTN, −353AMTN, −501AMTN, −769AMTN and −950AMTN, were increased by IL‐1β (1 ng·mL^−1^, 12 h) in Ca9‐22 cells. The results of transcriptional activities obtained from three separate transfections with constructs, pGL3‐basic and −100AMTN to −950AMTN, were combined, and values are expressed with standard errors. *P < 0.05 and **P < 0.01.

**Figure 5:**
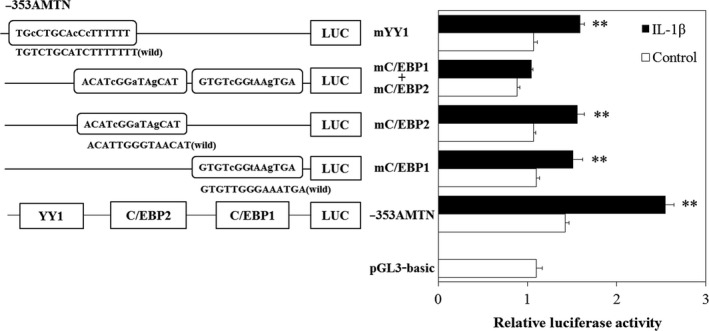
Site‐specific mutation analyses of LUC activities in Ca9‐22 cells. After introducing 3‐bp mutations, transcriptional induction by IL‐1β (1 ng·mL^−1^) was partially inhibited in the −353AMTNmC/EBP1, −353AMTNmC/EBP2, and −353AMTNmYY1. Double mutation in C/EBP1 and C/EBP2 (−353AMTNmC/EBP1 + mC/EBP2) almost completely abolished the effect of IL‐1β. The results of transcriptional activities obtained from three separate transfections with constructs were combined, and the values are expressed with standard errors. Significantly different from control, **P < 0.01.

**Figure 6:**
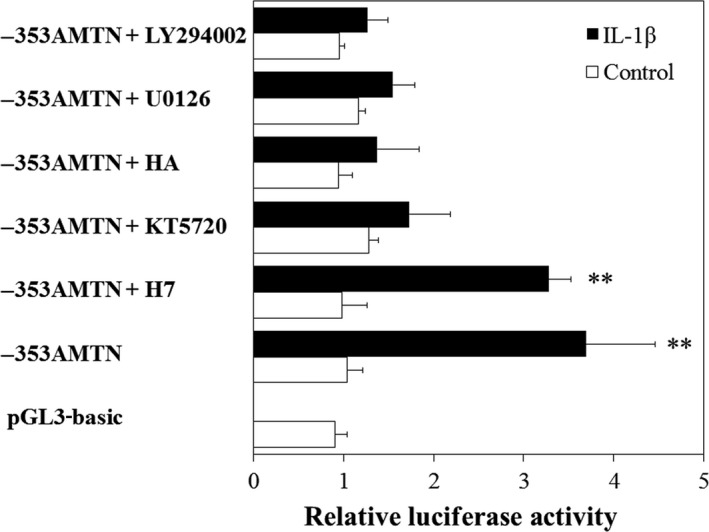
Effects of kinase inhibitors on transcriptional activities by IL‐1β. −353AMTN activities induced by IL‐1β were inhibited by KT5720, HA, U0126, and LY294002, and no effect was observed for H7. The results of transcriptional activities obtained from three separate transfections with constructs were combined, and values are expressed with standard errors. Significantly different from control, **P < 0.01.

The promoter sequence between −353 and +1 of the human AMTN gene contains an inverted TATA box (nts −21 to −12), an inverted CCAAT box (nts −67 to −63), an activator protein 1 (AP1; nts −94 to −84), a CCAAT/enhancer binding protein 1 (C/EBP1; nts −118 to −105), an octamer transcription factor 1 element (Oct1; nts −129 to −117), a C/EBP2 (nts −163 to −150), a Ying Yang 1 (YY1; nts −228 to −212), and a specificity protein 1 (SP1; −351 to −328; Fig. 3) 24. To determine the IL‐1β response regions in the human AMTN gene promoter, luciferase (LUC) constructs including various lengths of human AMTN gene promoter were transiently transfected into Ca9‐22 cells and their transcriptional activities were analyzed in the presence or absence of IL‐1β. The transcriptional activities of −211AMTN, −353AMTN, −501AMTN, −769AMTN, and −950AMTN were increased after 12‐h treatment with IL‐1β (1 ng·mL^−1^; Fig. 4). Transcriptional activity of −100AMTN was not induced by IL‐1β. IL‐1β‐induced LUC activity of −211AMTN was significantly higher than the activity of −100AMTN, and IL‐1β‐induced LUC activity of −353AMTN was significantly higher than the activity of −211AMTN (Fig. 4). Next, we prepared mutation constructs, −353AMTNmC/EBP1, −353AMTNmC/EBP2, −353AMTNmC/EBP1 + mC/EBP2, and −353AMTNmYY1, by introducing 3‐bp mutations in the putative response elements in −353AMTN construct. The basal transcriptional activities of all the mutation constructs were lower than the basal level of −353AMTN. Transcriptional inductions by IL‐1β (1 ng·mL^−1^) were partially inhibited in −353AMTNmC/EBP1, −353AMTNmC/EBP2, and −353AMTNmYY1 (Fig. 5). To confirm the functional elements, we also performed double‐mutation analyses using −353AMTNmC/EBP1 + mC/EBP2. The transcriptional activity of the construct with double mutation in C/EBP1 and C/EBP2 was further suppressed as compared with the construct introduced mutations only in C/EBP1 or C/EBP2 (Fig. 5). These results indicated that C/EBP1, C/EBP2, and YY1 are crucial for IL‐1β‐induced AMTN gene expression. We investigated the effects of protein kinase C (PKC) inhibitor H7, protein kinase A (PKA) inhibitor KT5720, tyrosine kinase inhibitor herbimycin A (HA), mitogen‐activated protein kinase (MAPK) kinase (MEK1/2) inhibitor U0126 and phosphatidylinositol 3‐kinase (PI3K) inhibitor LY294002 on the transcriptional activity of −353AMTN by IL‐1β. Whereas IL‐1β‐induced activity was inhibited by KT5720, HA, U0126, and LY294002, no effect was observed for H7 (Fig. 6).

### Gel mobility shift assays

**Figure 7:**
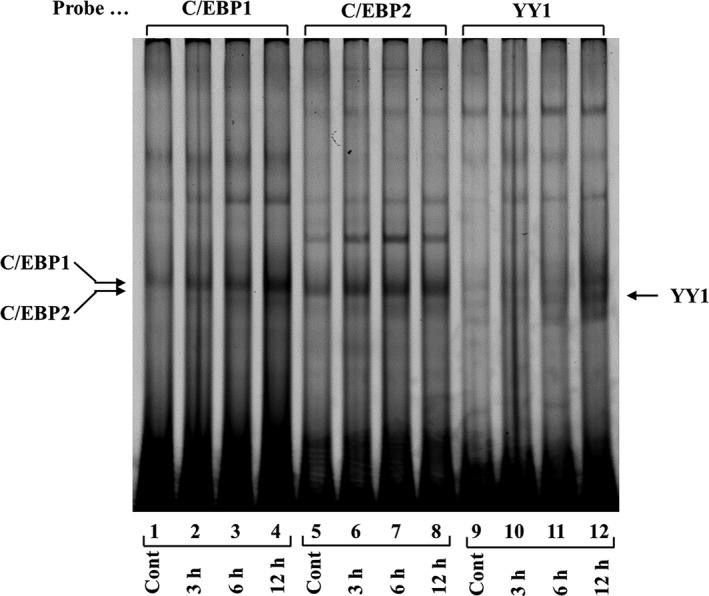
Gel mobility shift assays using C/EBP1, C/EBP2, and YY1. Cy5‐labeled double‐stranded C/EBP1, C/EBP2, and YY1 oligonucleotides were incubated with nuclear proteins obtained from Ca9‐22 cells stimulated with IL‐1β (1 ng·mL^−1^) for 3, 6, and 12 h. DNA–protein complexes were loaded on 6% PAGE and analyzed using an imaging system.

**Figure 8:**
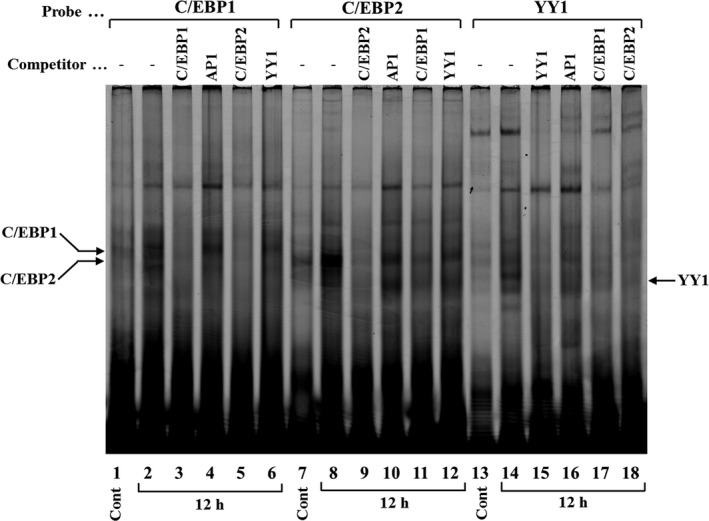
Specific binding of nuclear proteins to C/EBP1, C/EBP2, and YY1. Competition assays were performed using 40‐fold molar unlabeled oligonucleotides for AP1, C/EBP1, C/EBP2, and YY1. DNA–protein complexes were loaded on 6% PAGE and analyzed using an imaging system.

To identify the binding of nuclear proteins to the C/EBP1, C/EBP2, and YY1 elements, Cy5‐labeled double‐stranded oligonucleotides were incubated with equal amounts (4 μg) of nuclear proteins extracted from Ca9‐22 cells that were either not treated (control) or treated with IL‐1β (1 ng·mL^−1^) for 3, 6, and 12 h. After stimulation by 1 ng·mL^−1^ IL‐1β, C/EBP1–protein and C/EBP2–protein complexes were increased at 3 and 6 h and reached maximum at 12 h (Fig. 7, lanes 2–4, lanes 6–8). YY1–protein complex was increased at 6 h and reached maximum at 12 h (Fig. 7, lanes 11 and 12). These DNA–protein complexes represent specific interactions that were confirmed by competition gel shift assays using a 40‐fold molar excess of C/EBP1, C/EBP2, and YY1 nonlabeled double‐stranded oligonucleotides (Fig. 8, lanes 3, 9, and 15). AP1 did not compete with C/EBP1–protein, C/EBP2–protein, and YY1–protein complex formations (Fig. 8, lanes 4, 10, and 16). C/EBP2 and C/EBP1 oligonucleotides disrupted C/EBP1–protein and C/EBP2–protein complex formations, respectively (Fig. 8, lanes 5 and 11). These results demonstrate that the proteins binding to C/EBP1 and C/EBP2 elements are similar transcription factors. C/EBP1 and C/EBP2 almost completely competed with YY1–protein complex formation (Fig. 8, lanes 17 and 18), suggesting that the constituents of YY1‐binding proteins resemble C/EBPs.

### ChIP assays

**Figure 9:**
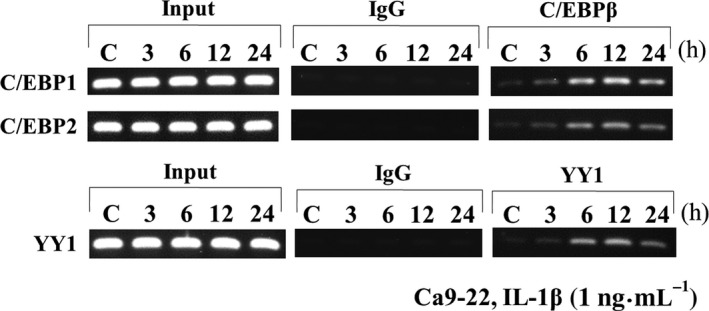
ChIP analyses of transcription factors binding to C/EBP1, C/EBP2, and YY1 in the human AMTN gene promoter in Ca9‐22 cells. PCR bands amplified and corresponding to DNA–protein complexes immunoprecipitated with antibodies showed that C/EBPβ and YY1 interacted with a chromatin fragment containing the C/EBP1, C/EBP2, and YY1, which were increased in Ca9‐22 cells following stimulation with IL‐1β for 3, 6, 12, and 24 h.

**Figure 10:**
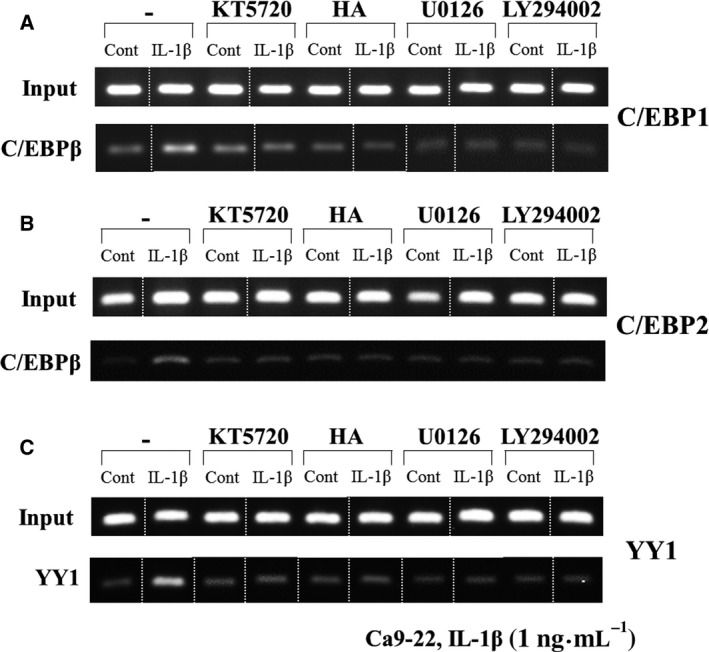
ChIP assays of transcription factors binding to C/EBP1, C/EBP2, and YY1 under the treatment by IL‐1β (1 ng·mL^−1^) with kinase inhibitors. Treatments with KT5720, HA, U0126, and LY294002 almost completely suppressed the induction of C/EBPβ and YY1 bindings to C/EBP1 (A), C/EBP2 (B), and YY1 (C) by IL‐1β for 12 h. White dotted lines in the figure are splice marks indicating removal of intervening lanes or separate gels.

To examine whether C/EBPβ and YY1 transcription factors are able to interact directly with human AMTN gene promoter and how IL‐1β regulates these transcription factors’ interactions with the C/EBP1, C/EBP2, and YY1, we performed ChIP assays. For these experiments, soluble chromatins were obtained from Ca9‐22 cells treated with IL‐1β (1 ng·mL^−1^) for 0, 3, 6, and 12 h and immunoprecipitated with either antibodies or control IgG. The PCR bands amplified and revealed that C/EBPβ interacted with a chromatin fragment containing the C/EBP1 and C/EBP2 which were increased after stimulation by IL‐1β at 3 h, reached maximum at 12 h, and decreased at 24 h (Fig. 9). YY1 interacted with a chromatin fragment containing the YY1 that was increased by IL‐1β at 6 and 12 h and decreased at 24 h (Fig. 9). Next, we examined how IL‐1β could regulate C/EBPβ bindings to C/EBP1 and C/EBP2, and YY1 bindings to YY1; four kinds of kinase inhibitors (KT5720, HA, U0126, and LY294002) were used with or without IL‐1β treatment. When Ca9‐22 cells were stimulated with IL‐1β for 12 h, KT5720, HA, U0126, and LY294002 almost completely abrogated C/EBPβ and YY1 bindings to C/EBP1, C/EBP2, and YY1 elements (Fig. 10A,B,C). These findings suggest that IL‐1β induced C/EBPβ and YY1 binding to C/EBP1, C/EBP2, and YY1 elements in the human AMTN gene promoter mediated through PKA, tyrosine kinase, MEK, and PI3 kinase pathways.

## Discussion

Inflammatory cytokines such as IL‐1β, tumor necrosis factor‐α (TNF‐α), and IL‐6 are soluble proteins that bind to specific receptors and induce intracellular signaling cascades 27. They play a fundamental role in inflammation including periodontal disease 24, 28. Having pivotal and wide‐ranging function in innate immunity and inflammation, IL‐1β regulates adaptive immunity and stimulates connective tissue turnover 29. In the present study, we have elucidated that IL‐1β induced AMTN gene transcription in Ca9‐22 cells by targeting C/EBP1, C/EBP2, and YY1 elements in the human AMTN gene promoter. We have previously reported that AMTN was highly induced in inflamed gingiva obtained from patients with chronic periodontitis 25, 26, and the results in this study have proven that the AMTN gene transcription was upregulated by inflammatory cytokine in gingival epithelial cells. IL‐1β production is initiated at early stage of inflammation 30 and plays a prominent role in the pathogenesis of periodontitis 31, and the concentration is increased in the periodontium 25, 26, 32. We have shown that AMTN gene expression is temporarily increased at the initiation of apoptosis by TGF‐β1 33. TNF‐α stimulates human AMTN gene transcription in gingival epithelial cells 24. AMTN protein expression in the JE was increased in *Porphyromonas gingivalis*‐infected periodontitis model mice at early stage, whereas AMTN protein levels were suppressed at later stages 34. These data suggest that the increased gene expression of AMTN by inflammatory cytokines might have some kind of physiological role of AMTN in the JE. AMTN could bind to itself and to ODAM, but not to other enamel proteins such as amelogenin (AMEL), ameloblastin (AMBN), and enamelin (ENAM). ODAM was found to interact with itself and with AMTN and AMBN and weakly with AMEL, but not with ENAM 35. More recently, it was shown that AMTN, ODAM, and secretory calcium‐binding phosphoprotein proline–glutamine‐rich 1 colocalized in the internal basal lamina of JE tend to interact with and among themselves and form supramolecular aggregates, and they participate in structuring an extracellular matrix with the distinctive capacity of attaching epithelial cells to mineralized surfaces 36.

Amelotin protein levels were increased by IL‐1β (1 ng·mL^−1^) after 3‐h stimulation and decreased at 24 h. CK19 protein levels were also decreased by IL‐1β at 24 h (Fig. 1C). CK19 expression was increased in inflamed gingival epithelium from patients with chronic periodontitis 37. The physiological means of increase or decrease of CK19 expressions are not clear; therefore, further study is necessary to dissolve these discrepancies.

From LUC analyses using various lengths of human AMTN gene promoter constructs (Fig. 4), we located the IL‐1β response regions of the proximal promoter of the human AMTN gene which encompasses C/EBP1, C/EBP2, and YY1 binding site (Fig. 3). IL‐1β upregulated LUC activities in ‐211, ‐353, ‐501, −769, and −950AMTN constructs, and the activity induced by IL‐1β was the highest at −353AMTN (Fig. 4). Transcriptional inductions by IL‐1β were partially abrogated when C/EBP1, C/EBP2, or YY1 elements were mutated in −353AMTN, and almost completely inhibited by double mutations in C/EBP1 and C/EBP2 (Fig. 5). The interaction between specific transcription factors and C/EBP1, C/EBP2, or YY1 elements with or without stimulation by IL‐1β was investigated by gel mobility shift assays (Fig. 7). These DNA–protein complex formations competed with 40‐fold molar excess of nonlabeled C/EBP1, C/EBP2, and YY1 elements (Fig. 8). C/EBP1–protein and C/EBP2–protein complexes competed with 40‐fold molar excess of nonlabeled C/EBP2 and C/EBP1. In addition, C/EBP1 and C/EBP2 almost completely competed with YY1–protein complex formations, suggesting that the constituents of YY1‐binding proteins resemble C/EBP‐binding proteins (Fig. 8). The results of ChIP assays indicated that IL‐1β induced AMTN gene transcription through C/EBPβ and YY1 transcription factors targeting to C/EBP1, C/EBP2, and YY1 elements in the human AMTN gene promoter (Fig. 9). C/EBPs are leucine zipper transcription factors that regulate various aspects of cellular differentiation and function in a variety of tissues 38. C/EBPβ was originally identified as a mediator of IL‐6 signaling, and signal transduction of the acute phase response by IL‐1, IL‐6, and lipopolysaccharide induces C/EBPβ transcription 39. YY1 is a ubiquitous and multifunctional zinc‐finger transcription factor of the Polycomb group protein family 40. TNF‐α and IL‐1β promoted YY1 expression in the fibroblast‐like synoviocytes of rheumatoid arthritis patients 41. We have identified C/EBPβ and YY1 as transcription factors that are important for human AMTN gene transcription regulated by IL‐1β in this study.

Interleukin‐1 receptors heterodimerize after cytokine binding. IL‐1α and IL‐1β bind to IL‐1R1 and use IL‐1RAcP as a common coreceptor and then recruit intracellular signaling molecules, including myeloid differentiation factor 88, IL‐1R‐associated kinase, and TNF receptor‐associated factor 6 (TRAF6) to activate nuclear factor‐κB, as well as extracellular signal‐regulated kinase 1/2, c‐Jun N‐terminal kinase, and p38 MAPK 42. To elucidate the signaling pathways which regulate AMTN gene transcription induced by IL‐1β, we performed LUC and ChIP assays using several kinds of kinase inhibitors (Figs 6 and 10). The PKA inhibitor KT5720, the tyrosine kinase inhibitor HA, the MEK1/2 inhibitor U0126, and the PI3K inhibitor LY294002 inhibited the effects of IL‐1β on AMTN gene transcription, suggesting that these signaling pathways are crucial for transcriptional regulation of AMTN gene by IL‐1β.

In conclusion, we have demonstrated that AMTN gene transcription was induced by IL‐1β in Ca9‐22 gingival epithelial cells, and characterized IL‐1β response elements in the human AMTN gene promoter as C/EBP1, C/EBP2, and YY1. IL‐1β regulates AMTN gene transcription via PKA, tyrosine kinase, MEK1/2, and PI3K pathways. Additionally, C/EBPβ and YY1 transcription factors appear to be key regulators of IL‐1β effects on AMTN gene transcription. These observations suggest that AMTN is increased in inflamed gingival epithelium and might have some physiological role in the inflamed JE.

## Materials and methods

### Reagents

Alpha‐minimum essential medium (α‐MEM), human recombinant IL‐1β, and tyrosine kinase inhibitor HA were purchased from Wako (Tokyo, Japan). FBS, penicillin and streptomycin, TrypLE Express, and Lipofectamine 2000 were purchased from Invitrogen (Carlsbad, CA, USA). ISOGEN II was purchased from Nippon Gene (Tokyo, Japan). PrimeScript RT reagent kit and SYBR Premix Ex Taq II were purchased from Takara‐Bio (Tokyo, Japan). pGL3‐basic LUC plasmid, pSV‐β‐galactosidase (β‐Gal) control vector, and MEK1/2 inhibitor U0126 were obtained from Promega (Madison, WI, USA). PKC inhibitor H7 was from Seikagaku Corporation (Tokyo, Japan). PKA inhibitor KT5720, complete protease inhibitor cocktail, and PMSF were purchased from Sigma‐Aldrich Japan (Tokyo, Japan). PI3K inhibitor LY294002 was from Calbiochem (San Diego, CA, USA). QuikChange Site‐Directed Mutagenesis Kit was purchased from Agilent Technologies (Santa Clara, CA, USA). Anti‐mouse IgG (whole molecule) peroxidase antibody produced in rabbit, anti‐rabbit IgG (whole molecule) peroxidase antibody produced in goat, and ECL Prime Western Blotting Detection Reagents were purchased from GE Healthcare (Buckinghamshire, UK). All chemicals used were of analytical grade.

### Cell cultures

Human gingival epithelial Ca9‐22 cells were cultured in α‐MEM containing 10% FBS at 37 °C in 5% CO_2_/95% air. Cells were grown to confluence in 60‐mm cell culture dishes and then cultured in α‐MEM without serum for 12 h. After that, Ca9‐22 cells were incubated with different concentrations of IL‐1β (0.1, 1, 10, and 50 ng·mL^−1^) for 12 h or 1 ng·mL^−1^ IL‐1β for 0 (control), 3, 6, 12, and 24 h. Total RNA was isolated from triplicate cultures using ISOGEN II and analyzed for the expression of AMTN and glyceraldehyde 3‐phosphate dehydrogenase (GAPDH) mRNA by real‐time PCR.

### Real‐time PCR

Total RNA (1 μg) was used as a template for cDNA which was prepared using the PrimeScript RT reagent kit. Quantitative real‐time PCR was performed using the following primer sets: AMTN forward, 5′‐GTTGAATGTACAACAGCAACTGCAC‐3′; AMTN reverse, 5′‐TTCCATCCTGGACATCTGGATTAG‐3′; GAPDH forward, 5′‐GCACCGTCAAGGCTGAGAAC‐3′; GAPDH reverse, 5′‐ATGGTGGTGAGACGCCAGT‐3′, using the SYBR Premix Ex Taq II in a TP800 Thermal Cycler Dice Real‐Time System (Takara‐Bio). The amplification reactions were performed in 25 μL of final volume containing ×2 SYBR Premix EX Taq (12.5 μL), 0.4 μm forward and reverse primers (0.2 μL), and 70 ng cDNA (7 μL) for AMTN and 50 ng cDNA (5 μL) for GAPDH. To reduce variability between replicates, PCR premixes containing all reagents except for cDNA were prepared and aliquoted into 0.2‐mL PCR tubes (NIPPON Genetics). The thermal cycling conditions were 10 s at 95 °C, 45 cycles of 5 s at 95 °C, and 30 s at 60 °C. Post‐PCR melting curves confirmed the specificity of single‐target amplification, and the expressions of AMTN relative to the GAPDH were determined in triplicate 24.

### Western blot

Total proteins extracted from Ca9‐22 cells were separated by 12% SDS/PAGE and transferred onto a Hybond 0.2‐μm PVDF membrane. The membrane was incubated with anti‐AMTN (ab122312; Abcam, Cambridge, UK), anti‐CK19 (ab7755; Abcam), and anti‐α‐tubulin (sc‐5286; Santa Cruz Biotechnology, CA, USA) antibodies for 2 h. Anti‐rabbit and anti‐mouse IgG conjugated with horseradish peroxidase were used as the secondary antibodies. Immunoreactivities were detected by ECL Prime Western Blotting Detection Reagents 24.

### Immunofluorescence

Eight‐chamber slides were coated with 10 μg·mL^−1^ fibronectin at 37 °C for 30 min. Ca9‐22 cells were plated on eight‐chamber slides at 10 000 cells·mL^−1^ and cultured in α‐MEM with 10% FBS for 12 h. Medium was changed to α‐MEM without serum for 6 h, and then, cells were treated with 1 ng·mL^−1^ IL‐1β for 6 h. After 6 h, cells were fixed in 4% paraformaldehyde for 10 min and then treated with 0.1% Triton X‐100 for 5 min for permeabilization. Cells were blocked in 2.5% goat serum in 4% bovine serum albumin for 20 min at room temperature. Primary antibody was rabbit polyclonal anti‐AMTN (ab122312; Abcam) used at 1 : 200 for 2 h at 37 °C. Secondary antibodies were Alexa Fluor 488 goat anti‐rabbit IgG used at 1 : 200 for 1 h at room temperature. Coverslips were mounted with AntiFade Poly/Mount with DAPI (Polysciences, Warrington, PA, USA). For analysis of the expression of AMTN by Ca9‐22 cells, cells were imaged using a LSM 5 EXCITER (Carl Zeiss Microscopy, Jena, Germany). Exposure time and intensity range were the same for each image. Contrast‐adjusted postimaging was equal for all images.

### Luciferase assays

To explore the IL‐1β response regions in human AMTN gene promoter, we prepared LUC constructs by ligating human AMTN gene promoters into pGL3‐basic vector. Various lengths of human AMTN gene promoter sequences (−100AMTN: −100 to +60; −211AMTN: −211 to +60; −353AMTN: −353 to +60; −501AMTN: −501 to +60; −769AMTN: −769 to +60; −950AMTN: −950 to +60) were prepared by PCR amplification, and these DNA were cloned into the SacI site of the pGL3‐basic multicloning site. Mutation C/EBP1 (−353AMTNmC/EBP1; GTGTcGGtAAgTGA), mutation C/EBP2 (−353AMTNmC/EBP2; ACATcGGaTAgCAT), double‐mutation C/EBP1 and C/EBP2 (−353AMTNmC/EBP1 + mC/EBP2), and mutation YY1 (−353AMTNmYY1; TGcCTGCAcCcTTTTTT) constructs were made by PCR using the QuikChange Site‐Directed Mutagenesis Kit within the context of the homologous −353 to +60AMTN promoter fragments 24.

Twenty‐four hours after plating, Ca9‐22 cells at 60–80% confluence were transfected by transfection mixture including 1 μg LUC construct and 1 μg β‐Gal plasmid as an internal control using Lipofectamine 2000. Two days after transfection, the cells were deprived of serum for 12 h, and IL‐1β (1 ng·mL^−1^) was added for 12 h prior to harvesting. The LUC assays were performed according to the supplier's protocol (PicaGene; Toyo Ink, Tokyo, Japan) using a luminescence reader (AccuFLEX Lumi 400; Aloka, Tokyo, Japan) to measure LUC activities. Several types of protein kinase inhibitors were used for protein kinase inhibition. Two days following transfection, the cells were deprived of serum for 12 h and first treated with H7 (5 μm), KT5720 (100 nm), LY294002 (10 μm), and U0126 (5 μm) for 30 min, or HA (1 μm) for 4 h, and then incubated with IL‐1β (1 ng·mL^−1^) for 12 h before harvesting.

### Gel mobility shift assays

Ca9‐22 cells were grown to confluence and then cultured in α‐MEM without serum for 12 h. After that, confluent Ca9‐22 cells incubated for 3, 6, and 12 h with IL‐1β (1 ng·mL^−1^) in α‐MEM without serum were used to prepare the nuclear extracts. The control Ca9‐22 cells were cultured in α‐MEM without serum for 12 h, and they were harvested without stimulation by IL‐1β. Double‐stranded oligonucleotides encompassing the 5′‐Cy5‐labeled C/EBP1, C/EBP2, and YY1 in the human AMTN gene promoter were used as DNA probes (Sigma‐Aldrich Japan), and they were annealed under optimal conditions (50 mm Tris/HCl pH 7.9, 10 mm MgCl_2_). Nuclear proteins (4 μg) were incubated for 20 min at room temperature with 2 pm Cy5‐labeled double‐stranded oligonucleotide in the binding buffer containing 50 mm KCl, 0.5 mm EDTA, 10 mm Tris/HCl (pH 7.9), 1 mm dithiothreitol, 0.04% Nonidet P‐40, 5% glycerol, and 1 μg of poly(dI‐dC). After incubation, the DNA–protein complexes were separated by 6% nondenaturing acrylamide gels run at 200 V. Following electrophoresis, the gels were analyzed using a Typhoon TRIO+ Variable Mode Imager (GE Healthcare). For competition experiments, 40‐fold molar unlabeled oligonucleotides of AP1, C/EBP1, C/EBP2, and YY1 were used. The double‐stranded oligonucleotide sequences were as follows: C/EBP1 For: 5′‐AATGAAGTGTTGGGAAATGAAAAAAAA‐3′, C/EBP1 Rev: TTTTTTTTCATTTCCCAACACTTCATT‐3′; C/EBP2 For: 5′‐TGGTAGACATTGGGTAACATAATGTC‐3′, C/EBP2 Rev: 5′‐GACATTATGTTACCCAATGTCTACCA‐3′; and YY1 For: 5′‐TATTGTCTGCATCTTTTTTTAATTA‐3′, YY1 Rev: 5′‐TAATTAAAAAAAGATGCAGACAATA‐3′ 24.

### ChIP assays

ChIP assays were performed as described previously 24. The purified DNA were used for PCR amplification (1 cycle at 95 °C, 3 min, amplification was carried out for 35 cycles, denaturing at 95 °C, 15 s; 55 or 57 °C, 15 s; 72 °C, 1 min; and final extension at 72 °C, 1 min) for the C/EBP1, C/EBP2, and YY1 site within the human AMTN promoter using C/EBP1 ChIP For: 5′‐ AATGTCTGCAGCTAATAACC‐3′, C/EBP1 ChIP Rev: 5′ GGAAACTGAGAGACCAATCA‐3′; C/EBP2 ChIP For: 5′‐AATTACATAAGGCCTGTCTCC‐3′, C/EBP2 ChIP Rev: 5′‐ TTCATTAGGTGGGTTATTAGC‐3′; and YY1 ChIP For: 5′‐ GAAGAAGGAGGTGAAGAAGC‐3′, YY1 ChIP Rev: 5′‐ CTACCATTTGAATAGAAGGTG‐3′. KAPA Taq™ EXtra HotStart was utilized for the PCR procedure, and the PCR products were separated on 2% agarose gels and visualized with ultraviolet light.

### Statistical analysis

Triplicate samples were analyzed for each experiment, and experiments were replicated to ensure the consistency of the responses to drugs. Significant differences between control and treatment groups were determined using the one‐way ANOVA.

## Author contributions

We declared that all the listed authors have participated actively in the study and all meet the requirements of the authorship. MY and YO designed the study, wrote the protocol, undertook the statistical analysis, and wrote the first draft of the manuscript. MY, MM, KN, and YI performed research/study. SM, HT, and YN managed the literature searches and analyses.
